# Supplementary material for: A high-resolution mRNA expression time course of embryonic development in zebrafish
Source: eLife. 2017 Nov 16;6:e30860. doi: 10.7554/eLife.30860 (PMC5690287; doi:10.7554/eLife.30860)
Supplement: Supplementary file 6. [file elife-30860-supp6.zip › biolayout-clusters-files/Cluster251.html]

Cluster251


# Cluster251: Detail

### Go to ZFA detail

## GO

| GO ID | Description | Domain | Annotated | Expected | Observed | Adjusted p-value | Genes | Ensembl IDs |
| --- | --- | --- | --- | --- | --- | --- | --- | --- |
| GO:0000462 | maturation of SSU-rRNA from tricistronic... | biological\_process | 21 | 0.01 | 2 | 0.0086 | diexf rrp36 | ENSDARG00000017696 ENSDARG00000032516 |
| GO:0005730 | nucleolus | cellular\_component | 111 | 0.07 | 3 | 0.0056 | diexf rrp36 nol12 | ENSDARG00000017696 ENSDARG00000032516 ENSDARG00000038027 |
| GO:0019843 | rRNA binding | molecular\_function | 26 | 0.01 | 2 | 0.0122 | diexf nol12 | ENSDARG00000017696 ENSDARG00000038027 |
